# Association of Editors

**Published:** 1856-04

**Authors:** 


*Association of Editors.*

We copy the following from the April number of the *Philadelphia Medical and Surgical Journal.*

We think an Association of the kind proposed, might be made very useful, and are ready to co-operate in any reasonable movement for effecting its organization:—

"It has been proposed, during several past years, by some of the editors of the medical journals of this country, to form an association of editors; which, by uniting the energies of the medical press upon given subjects calculated to elevate the profession, would be more effective in accomplishing the purposes which an independent and high-toned press could not so readily attain by divided and individual action. This matter has been pressed upon the attention of the profession from year to year without as yet resulting in the object in view. It is to be hoped that the members of the medical press who will meet at Detroit the coming spring in the American Medical Association, will take measures to effect the proposed organization. The only obstacle which we can perceive to this movement, is the fact, that a large proportion of the medical journals of the country, are supposed to represent certain local, private interests, which may conflict with the general interests of the profession. We do not think, however, that there is any *real*

ground for this assumption. In promoting the interests of any locality there need be no conflict with those of the great body of the profession. Let individuals and cliques be forgotten, and the organization will be easily accomplished."

